# Electromagnetic Biostimulation of Living Cultures for Biotechnology, Biofuel and Bioenergy Applications

**DOI:** 10.3390/ijms10104515

**Published:** 2009-11-20

**Authors:** Ryan W. Hunt, Andrey Zavalin, Ashish Bhatnagar, Senthil Chinnasamy, Keshav C. Das

**Affiliations:** 1 Department of Biological and Agricultural Engineering, The University of Georgia, Athens, GA 30602, USA; E-Mails: bhatnagarashis@gmail.com (A.B.); csenthil@engr.uga.edu (S.C.); kdas@engr.uga.edu (K.C.D.); 2 Mass Spectrometry Research Center, Vanderbilt University Medical Center, Nashville, TN 37232, USA; E-Mail: andrey.zavalin@vanderbilt.edu (A.Z.)

**Keywords:** algae, bioenergy, biofuels, biomass, biostimulation, electromagnetic field, growth, metabolism, multipolar

## Abstract

The surge of interest in bioenergy has been marked with increasing efforts in research and development to identify new sources of biomass and to incorporate cutting-edge biotechnology to improve efficiency and increase yields. It is evident that various microorganisms will play an integral role in the development of this newly emerging industry, such as yeast for ethanol and *Escherichia coli* for fine chemical fermentation. However, it appears that microalgae have become the most promising prospect for biomass production due to their ability to grow fast, produce large quantities of lipids, carbohydrates and proteins, thrive in poor quality waters, sequester and recycle carbon dioxide from industrial flue gases and remove pollutants from industrial, agricultural and municipal wastewaters. In an attempt to better understand and manipulate microorganisms for optimum production capacity, many researchers have investigated alternative methods for stimulating their growth and metabolic behavior. One such novel approach is the use of electromagnetic fields for the stimulation of growth and metabolic cascades and controlling biochemical pathways. An effort has been made in this review to consolidate the information on the current status of biostimulation research to enhance microbial growth and metabolism using electromagnetic fields. It summarizes information on the biostimulatory effects on growth and other biological processes to obtain insight regarding factors and dosages that lead to the stimulation and also what kind of processes have been reportedly affected. Diverse mechanistic theories and explanations for biological effects of electromagnetic fields on intra and extracellular environment have been discussed. The foundations of biophysical interactions such as bioelectromagnetic and biophotonic communication and organization within living systems are expounded with special consideration for spatiotemporal aspects of electromagnetic topology, leading to the potential of multipolar electromagnetic systems. The future direction for the use of biostimulation using bioelectromagnetic, biophotonic and electrochemical methods have been proposed for biotechnology industries in general with emphasis on an holistic biofuel system encompassing production of algal biomass, its processing and conversion to biofuel.

## 1. Introduction

Electromagnetic fields are capable of eliciting *in vivo* and *in vitro* effects in many biological systems [1]. Increasing attention is being directed towards bioelectromagnetic stimulation of living cultures for biotechnology and bioenergy applications using the low frequency electromagnetic fields (EMF). A number of bioprocesses could be successfully integrated with electromagnetic or electrochemical stimulation if the cultivation conditions are properly engineered using specialized reactors viz. electrolytic bioreactors, electro-bioreactors and bioelectro-reactors [2]. Most recently, a strong initiative in bioenergy research has been taken up to investigate methods for enhancing productivity and metabolic processes for biomass production and biorefining of biomass for production of biofuels, energy and other added value products. Currently, microalgae are considered to be the most promising candidates for biomass production because of their ability to grow fast, produce large quantities of lipids, carbohydrates and proteins, thrive in poor quality waters, sequester and recycle carbon dioxide from industrial flue gases and remove pollutants from industrial, agricultural and municipal wastewaters. Microalgae are novel feedstocks for renewable biomass production that is capable of meeting the global demand for transportation fuels because the oil productivity of many strains of microalgae greatly exceeds that of the most productive oil crops such as oil palms and soybean [3]. Although biomass production may be most effectively performed by large-scale algae cultivation, yeast and bacteria are the most common groups of organisms used in bioprocessing and conversion technologies like fermentation, composting, anaerobic digestion and bioremediation. Considering the current importance of waste management and recycling in conserving natural resources, bioenergetic stimulation technologies may be used as a potential tool for bioremediation by stimulating the uptake rates of various polluting components found in the waste streams by microbes.

Extensive studies have been conducted over both eukaryotic (algae, yeasts and molds) and prokaryotic microorganisms using various electromagnetic regimes. The biological effects have been found to depend on field strength, frequency, pulse shape, type of modulation, magnetic intensity, and length of exposure [4]. Some results have been difficult to replicate due to various hidden parameters typically not monitored, such as local intensity and orientation of Earth’s geomagnetic field, cosmic radiations, solar winds and sunspot events.

Electromagnetism may affect organisms in both negative and positive manner which includes acceleration of growth and metabolism. This paper however focuses on the facilitative effects of electromagnetism on various microorganisms. The research attempts in this area can be divided into several groups based on implemented EMF parameters. Simplest initial classification can be based on time behavior of EMF and relative representation of the electric and magnetic components of the field. As it follows from the recent research results, a spatial configuration and topology of the EMF may also have significant impact on processes in living cultures. This paper also summarizes our own data regarding the effects of multipolar electromagnetic influences on biological systems and the future potential biostimulation techniques for improving microalgae biomass and lipid productivity for producing biofuels.

## 2. Electromagnetic Experiments

**Figure 1.**
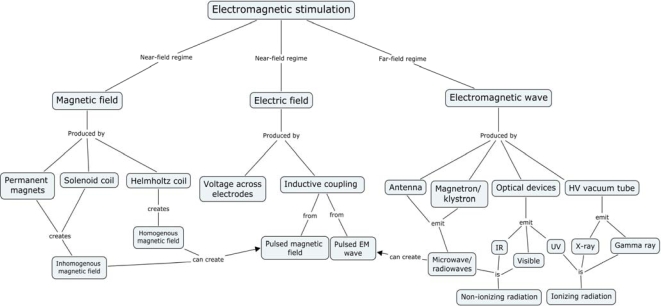
Overview of various electromagnetic stimulation modalities from fields and waves.

Three primary classes of experiments of electromagnetic influence (Figure 1) can be distinguished viz.:

- Predominantly magnetic fields: Near-field regime (Permanent, slowly changing, and pulsed fields from magnetic coils)
- Predominantly electric fields: Near-field regime (Permanent or slowly changing)
- Fields with both electric and magnetic components, with ratios between 0.1 and 10: Far-field regime (typical EMF oscillation frequency is 100 kHz or more)
- Fields from (I, II, or III) with unique spatial and/or temporal topology

Group I is represented relatively larger, mostly because of simplicity of experimental setup and extended penetration depth of magnetic field inside the water containing systems (Figure 4). The generated fields are either static magnetic fields or oscillating magnetic fields created by either permanent magnets or electromagnets, like Helmholtz and Solenoid coils. The biological experiments generally use a standard bipolar configuration with a N/S magnetic or +/− electric field for stimulation.

Group II is most often used in electroporation where strong pulsed electric fields (or PEF’s) are used for reversible membrane permeabilization to induce the uptake or release of some cell ingredients or foreign molecules. Group III is electromagnetic energy that propagates as a wave at higher frequencies and is considered as the far-field regime via an antenna, magnetron, or klystron. This classification encompasses non-ionizing radiowaves and microwaves, as well as optical and ionizing radiations such as IR, visible, UV, X-ray and Gamma radiation.

The following section on the effects of electromagnetic fields has been organized by the type of the EM treatment and further categorized on the basis of growth and physiological processes that have been studied within each treatment group.

## 3. Biostimulation by Electromagnetic Fields

### 3.1. Group I: Treatments Involving Magnetic Field Predominance

Experiments involving a predominant magnetic field have been conducted on a range of microorganisms that represent both prokaryotes (eubacteria, archaea) and eukaryotes (algae, fungi, protozoa). A wide variety of responses involving magnetic field predominance have been summarized in Table 1.

#### 3.1.1. Growth

Growth is a physiological response of an organism and a positive effect on growth indicates that some of the biosynthetic pathways are being stimulated. Erygin *et al.* [15] grew a gram-positive bacterium *Bacillus mucilaginous* in a magnetic field of ~0.26 T under different media compositions and compared it with unexposed control cultures. The magnetically treated liquid medium consisting of ferromagnetic salts showed rapid growth of the bacterium over control in 3 h. Similarly magnetically treated dry whey medium yielded three times higher cell count than the untreated medium. However, there was an overall increased response from the exposed dry whey illustrating how the culture medium composition may influence the effect of magnetic field.

Moore [17] studied five strains of bacteria and a yeast under a magnetic flux of 5–90 mT and reported maximum stimulation of growth at 15 mT (at 0.3 Hz) and maximum inhibition at 30 mT. Experiments with varying time especially using oscillating magnetic fields have uncovered new effects related to resonant phenomena in the living systems. The biostimulation of a denitrifying gram-negative bacterium *Pseudomonas stutzeri* by a magnetic field of 0.6–1.3 mT pulses via inductively coupled Helmholtz coils for 8–10 h resulted in a proliferation of biomass that was 10–30% more than the control [18].

Other than the medium conditions, magnetic flux and type of magnetic field, the exposure time is another major factor that governs the intensity of response. Justo *et al*. [8] observed that the growth of *Escherichia coli* could be stimulated or inhibited by exposure to an oscillating 100 mT extremely low frequency (ELF) magnetic field for 6.5 h. Exposed cells had 100 times greater viability than unexposed cells, however the viability varied with duration of exposure. It was suggested that the effect was a result of magnetic field driven alteration of membrane permeability and availability of ions in the culture medium.

Research groups in Japan and China have focused on investigating ways to improve the cultivation of the cyanobacterium *Spirulina platensis* for production of nutraceuticals using permanent magnetic fields. Hirano *et al.* [26] reported a significantly higher specific growth rate of 0.22 d^−1^ in *S. platensis* exposed to 10 mT magnetic field when compared to 0.14 d^−1^ for untreated culture. The growth of *S. platensis* was maximum when it was cultured phototrophically at lower light intensities; but did not show improvement under heterotrophic conditions.

Magnetic field induced growth stimulation in *S. platensis* has also been reported by Li *et al*. [27]. They observed a 47% increase in dry biomass on the sixth day of cultivation, and a 22% increase over control by day eight under the exposure of a 250 mT homogeneous magnetic field from a Helmholtz coil.

*Chlorella vulgaris* is another algal strain of interest for its nutraceutical value and is a promising producer of starch-glucose. This microalga can yield starch to the tune of 60 t ha^−1^ yr^−1^ which is 7.7 times more than that of traditional corn [50]. Takahashi *et al.* [31] used magnetic flux densities of 6–58 mT for cultivating *Chlorella* sp. and obtained facilitative growth at 40 mT. The specific growth rate of *Chlorella vulgaris* almost doubled from 0.07 to 0.12 d^−1^ under magnetic field generated using a dual-yoke electromagnet, which concentrates a magnetic field into a small cross-sectional area [30].

The static magnetic field strengths ranging from 0 to 230 mT on *Dunaliella salina* were used by Yamaoka *et al.* [32]. They observed an improvement in growth rate that peaked at 10 mT with the addition of 1 mg L^−1^ of Fe-EDTA. A ~0.26 T magnetic field exposure using different growth media for the yeast *Saccharomyces fragilis* showed that rapid growth (27–36% over the control in 3 h) occurred on magnetic treatment when a dry whey nutrient medium was used, but it turned inhibitory on using a liquid nutrient medium [15]. On the other hand Fiedler *et al.* [36] used an oscillating magnetic field generated by a Helmholtz coil via inductive coupling to produce 0.28–12 mT magnetic field at 50 Hz for 9 h to treat *S. cerevisiae*. They observed a maximum growth of 8 g L^−1^ of biomass under 0.5 mT magnetic field exposure and 6.8 g L^−1^ of biomass for the cells untreated.

#### 3.1.2. Photosynthesis and Cell Constituents

Hirano *et al*. [26] observed acceleration of the rates of O_2_ evolution as well as synthesis of sugar during photosynthesis in *Spirulina platensis* when exposed to 10 mT geomagnetic field. They opined that the treatment using magnetic field increased the phycocyanin content in *S. platensis,* which plays an important role in the activation of photosystem II to help the activation of electron transfer reactions during photosynthesis. Their results also suggested that the magnetic fields accelerate the light excitation of chlorophyll radical pair.

Li *et al.* [27] subjected the same cyanobacterium *S. platensis,* to a range of static magnetic field intensities among which some stimulated its growth, uptake of carbon and light energy utilization. They observed that the levels of micro and trace elements (Ni, Sr, Cu, Mg, Fe, Mn, Ca, Co and V) and essential amino acids such as histidine improved at 250 mT magnetic field treatments. Also, chlorophyll *a* content of the magnetically treated sample was higher than the control, suggesting better light harvesting for photosynthesis. However there was slight decrease in lipid synthesis.

In *Dunaliella salina,* β-carotene content could be raised when treated with 10–23 mT of static magnetic field and the maximum was obtained at 10 mT with addition of 1 mg L^−1^ of Fe-EDTA. It also showed higher accumulation of the heavy metals viz. Co, Cd, Cu and Ni in the magnetically treated cultures, indicating its potential for bioremediation of heavy metals [32].

Singh *et al.* [29] investigated the use of permanent magnets and found that the physiological response of a cyanobacterium *Anabaena doliolum,* was dependent on exposure time and magnetic pole orientation. They reported that N, S and N+S poles from 0.3 T permanent magnets produced different effects depending on the exposure time from 1 to 6 h. The effect was significant on a two hour exposure with combined N+S poles, where one culture was exposed to only N pole, which was then mixed with another culture exposed to S pole only. Treated cultures recorded 150, 110, 38, 34 and 20% increase in phycocyanin, chlorophyll *a*, carbohydrates, carotenoid and protein content, respectively and 55% increase in optical density over the control.

#### 3.1.3. Other Physiological Processes

##### 3.1.3.1. Ethanol Fermentation

The biotechnology of fermentation using yeasts, like *Saccharomyces cerevisiae,* has a long history in many sectors of industry from alcoholic beverages to ethanol production. A vital focus of ongoing research is the study of the key enzymes responsible for the production of the metabolites of interest, namely ethanol. Increasing the activity of key enzymes, like alcohol-dehydrogenase, is a primary goal of metabolic and enzyme engineering. The glucose dehydrogenase and alcohol dehydrogenase were studied in *S. cerevisiae* under the influence of a non-uniform pulsed magnetic field of 30 mT for 60 minutes [34]. They found that in the presence of NAD the glucose dehydrogenase activity increased 18%, while no effect was observed in the absence of NAD or NADP. The activity of alcohol dehydrogenase in the absence of co-enzymes rose to 10.7% in the anaerobically cultivated cells and 19.9% in those cultivated aerobically. The activity of this enzyme increased by 20.5% when NAD was added to this enzyme in the aerobic culture, while an 8.5% decrease was observed in the anaerobic culture. Thus, the non-homogenous pulsed magnetic field of 30 mT stimulated the activity of the dehydrogenases, but behaved differently in the absence or presence of NAD and NADP.

The effects on ethanol fermentation by *S. cerevisiae* under the influence of two styles of oscillating magnetic fields were studied by Perez *et al.* [35]. The primary magnetic field generator was composed of several permanent magnets stacked in series, while the recirculating culture broth was directed through the intervening space of the magnetic fields where spatial orientation determined the desired intensity of 5–20 mT for each exposure. The recirculation velocity passing through the array of static magnets modulated the frequency. The secondary generator was a double layer solenoid coil that produced 8 mT. Two magnetic field generators were coupled to the bioreactor, which were operated conveniently in simple or combined ways. The overall volumetric ethanol productivity enhanced by 17% over control at an optimum magnetic field treatment of 0.9–1.2 m s^−1^ velocity and 20 mT plus 8 mT solenoid. These results made it possible to verify the effectiveness of the dynamic magnetic treatment since the fermentations with magnetic treatment reached their final stage, 2 h earlier than the control. Perez *et al.* [35] postulated that membrane permeability and the redox system that are affected by the electromagnetic field might have resulted in alterations of ion transport of the substrates. As a consequence, the cellular metabolism was stimulated for higher ethanol production.

##### 3.1.3.2. Anti-Oxidant Defense System

Wang *et al.* [30] used a magnetic field concentrated to a small area and observed that it helped to regulate the anti-oxidant defense system of *Chlorella vulgaris* at a threshold magnetic flux intensity of 10–35 mT. The authors proposed that this is probably due to the free radicals altered by the magnetic field, which accelerated the relative biological reactions. The analysis of hydroxyl radical (^−^OH) showed that it increased simultaneously with increasing magnetic flux density suggesting an oxidative stress induced by the exposure compared to the control.

##### 3.1.3.3. Biodegradation

A study using airlift reactors showed that the influence of magnetic fields enhanced the degradation of phenolic waste liquors by submersed microorganisms at a magnetic field intensity of 22 mT [23].

#### 3.1.4. Genetic Machinery and Molecular Mechanisms

*E. coli* cells when placed under extremely low frequency (ELF) magnetic field sine wave of 30 μT at 9 Hz, exhibited a change in the conformational state of the genome, which was maximum at 4 × 10^8^ cells mL^−1^ while there was no such response at lower cell densities of 3 × 10^5^ cells mL^−1^. Other than cell density, time of exposure also affected genomic conformation. The change in the conformational state of the genome is considered to be dependent on DNA parameters, *i.e.* molecular weight and the number of proteins bound to the DNA [9]. Thus the ELF field which is close to the ion cyclotron resonance parameters for a medium weight ion might be influencing these factors that ultimately elicit response on the conformation. It was also proposed that the possibility of a resonance fluorescence effect where recombination of fluorescing radicals may act as signals for intercellular communication and participate in the synchronization of gene expression. Weak, static magnetic fields (0 110 μT) are shown affecting DNA-protein conformations in *E. coli.* The analysis by Binhi *et al.* [51] represented a dose-response curve for the static magnetic field. The curve however is peculiar in having three prominent maxima unlike other dose-response curves in nature that usually follow rising or decaying exponential functions. They explained this peculiarity in the context of the ion interference mechanism. No alteration in the profile of stress proteins of *E. coli* was observed by Nakasono *et al.* [52] on exposure to AC fields (7.8 14 mT, 5 100 Hz). In *Saccharomyces cerevisiae* no changes were observed under AC magnetic fields (10 300 mT, 50 Hz) in differential gene expression and protein profile that were determined using microarray and 2-D protein profile analysis, respectively [53]. But, Gao *et al.* [54] reported that strong magnetic fields (14.1 T) could lead to transcriptional up-regulation of 21 genes and down-regulation of 44 genes in a gram-negative anaerobic bacterium *Shewanella oneidensis* that did not show any significant effect on growth. In the anoxygenic photosynthetic bacterium, *Rhodobacter sphaeroides*, AC magnetic fields of 0.13−0.3 T induced a 5-fold increase in porphyrin synthesis, and enhanced expression of the enzyme 5-amino-levulinic acid dehydratase, while very strong DC fields (0.13−0.3 T) also induced synthesis of this enzyme predominantly at the magnetic North Pole. The effects are attributed to elevated gene expression that ultimately resulted in increased porphyrin production [25].

Mitotic delay of 0.5 to 2 h was observed in a slime mold *Physarum polycephalum* in presence of ELF electromagnetic fields (45, 60 and 75 Hz) by Goodman *et al.* [44]. Removal of the mold from magnetic field recovered normal mitosis in 40 days.

### 3.2. Group II: Treatments Involving Electric Field Predominance

Stimulation in the growth of immobilized *E. coli* cells by 140% over control, was reported by Chang *et al.* [10], which was attributed to the enhanced removal of inhibitory products from the gel through electro-osmosis and electrophoresis as well as an augmented glucose supply.

Kerns *et al.* [19] reported growth stimulation in *Trichoderma reesei* by using pulsed EMF’s for electroporation via inductively coupled electric currents from a Helmholtz coil. The use of electric fields has also been investigated with yeasts in either a static mode or an oscillating/pulsed mode. The survival rate of *Saccharomyces cerevisiae* was investigated under bipolar electric field pulses from 0–1.5 kV/cm by measuring plating efficiency. The maximum growth after plating appeared at 0.85 kV/cm which demonstrated a 100% increase over the control [55]. An electrostimulation in *S. cerevisiae* from electric field application at 10 mA DC and 100 mA AC resulted in an increase in growth rate by 60% in AC mode and 50% in DC along with an increase in the production of the acetic acid, lactic acid and acetaldehyde. The results suggest that the acceleration of growth rate from a DC exposure stimulated cell budding during the early stages of cultivation, which could be due to a 60% decrease in inhibitory concentration of dissolved CO_2_ and other chemical modifications of the culture medium [38]. Zrimec *et al.* [12] have shown that external AC electric fields of low intensity stimulated membrane bound ATP synthesis in starving *E. coli* cells with electric field amplitudes of 2.5–50 V/cm and a frequency optimum at 100 Hz. The model of electro conformational coupling was used to analyze the frequency and amplitude responses of ATP synthesis. Two relaxation frequencies of the system were obtained at 44 and 220 Hz, and an estimate of roughly 12 elementary charges was obtained as the effective charge displacement for the catalytic cycle of ATP synthesis.

An actinomycetous eubacterium *Streptomyces noursei* used for antibiotic production was electrostimulated via PEMF’s using a pair of Helmholtz coils via inductive-coupling producing 5 ms bursts of 220 μs duration in intervals of 60 ms by Grosse [20]. The process resulted in a mean inductive electric field strength of approximately 1.5 mV cm^−1^. An increase was observed in the formation of the product but only during the first 50 hours of the starting phase although the exposed culture exhibited an overall increase in O_2_ consumption and glucose utilization.

Electric field stimulation may also be used to improve the substrate utilization efficiency in microbial processes. Cells when subjected to electric field pulses of 0.25 kV for 10 ms in the presence of the enzyme cellobiose showed enhanced utilization of cellobiose and conversion of substrate into ethanol by a thermotolerant yeast, *Kluyveromyces marxianus.* As a result, ethanol yield increased by nearly 40% over the control [43].

Kerns *et al.* [19] showed that pulsed EMF’s at 1.5 mVcm^−1^ bursts for 115 hours used for electroporation lead to ~60% increase in cellulase activity and ~80% increase in cellulase secretion in *Trichoderma reesei* . They concluded that the effect occurred inside the cells on either the formation of the cellulase enzyme complex at the genetic level or the secretion into the medium via altered membrane permeability.

A 62% increase in biosorption of uranium was observed using pulsed electric fields of 1.25 to 3.25 kV cm^−1^, suggesting that the application of short and intense pulses might enhance the biosorption of toxic metals and radionuclides from wastewater streams [24].

### 3.3. Group III: Treatments Involving both Electric and Magnetic Fields in Far-Field Regime

Some of the original pioneering work with the bioeffects from weak electromagnetic radiation in the form of microwaves was performed in Russia and extended into Europe in the 1970’s. The work by Grundler *et al.* [39], investigated the use of very weak microwave irradiation of a few mW/cm^2^ at a frequency around 42 GHz ±10 MHz on *Saccharomyces cerevisiae.* The experiments demonstrated multiple resonance dependent effect of coherent millimeter electromagnetic waves in the frequency region of 41.83 to 41.96 GHz that increased growth rates up to 15% or decreased the growth rate by 29% depending on frequency.

Banik *et al.* [5] investigated the use of electromagnetic irradiation at the microwave frequency from 13.5 to 36.5 GHz on *Methanosarcina barkeri* DSM-804, a methanogenic archaebacterium used in anaerobic digestion for biogas production. The bacteria were exposed for 2 h duration for three days before inoculation into the anaerobic digesters. Significant increases in methane (CH_4_) concentration were observed that peaked at 76.3% CH_4_ at 31.5 GHz, compared to 52.3% CH_4_ in control. Furthermore, an increase in specific growth rate was observed for every frequency with a significant reduction in the lag phase. The irradiated cultures had higher cell numbers and the cell diameter was enlarged by 20%. It was concluded that the growth rate and biomethanation potential of *M. barkeri* DSM-804 could favorably induce catalytic abilities via a thermal microwave irradiation at 31.5 GHz.

Tambiev and co-workers (cited in [28]) observed that exposure of high frequency microwaves for 30 min at 2.2 mW cm^−2^ and 7.1 mm wavelength enhanced the growth of the cyanobacterium *Spirulina platensis* by 50%. Belyaev *et al.* [56] suggested that there was frequency-specific resonant interaction between low-intensity microwave and chromosomal DNA in *E. coli.*

### 3.4. Group IV: Treatments with Spatial/Temporal Topology

#### 3.4.1. Spatial Superposition

The investigation of using multiple independent field sources has led to studies where the treatment area exhibits spatial topology from superposition. A magnetic therapeutic device that uses four nonuniform static magnets in four-pole symmetry demonstrates an increased rate of Myosin phosphorylation over control. The notion that the magnetic field amplitude is the only parameter involved to determine the outcome with magnetobiology experiments has been shown to be false and it is suggested the topological parameters in a spatial domain, such as field gradient and symmetry might also be of relevance [57].

Mazur investigated the use of multiple magnetic fields in superposition on biological samples. He exposed *S. cerevisiae* to a six-pole electromagnet with coils of alternating polarity at a magnetic field of 0.39–0.52 T, while saturating it with pure molecular oxygen. The magnetic field has an influence on the biosynthesis of yeast and changes their enzymatic activity when grown under aerobic conditions as opposed to anaerobically cultivated yeast. He found that in the presence of a magnetic field, the oxygen saturation increased from 5.37 to 39.9 mg L^−1^ and simultaneously stabilized the pH. The initiation of fermentation occurred immediately after mixing of the dough. It was found that there was an improvement in the physical qualitative property of rising strength, which was decreased from 76 minutes to 53 minutes in the presence of oxygen saturation and a magnetic field. It was also found that the increase in CO_2_ production was 3.7 times greater in the magnetic treated culture than the control, which indicates a significant increase in maltase activity. The amount of dissolved oxygen in water increased and was sharply activated in the presence of a magnetic field [58].

#### 3.4.2. Spatial and Temporal Superposition

Aspects of EMF topology in the time domain have been studied by researchers looking at the influence on biological systems from combined AC and DC EMFs in superposition [58–61]. It has been shown that the cellular response to the orientation of the fields is distinct depending whether the AC and DC fields are perpendicular and parallel to each other. It was found that the perpendicular orientation is dominant in an intensity-dependent non-linear manner [61]. There is a fundamental difference in the spatial pattern of cellular response between DC and pulsed stimulation [62]. Several studies report that the relative orientation of AC and DC magnetic fields appears to be critical for a number of calcium-dependent cell processes. The data suggests that DC magnetic fields influence biological membranes in a somewhat different manner than low frequency AC magnetic fields [1].

#### 3.4.3. Multipolar Electromagnetic Systems

The advent of quantum theories on the molecular scale has inspired the development of electromagnetic exposure systems that mimic the complex interactions and symmetry found in nature from endogenous electromagnetic signals and their destructive interference between interdependent cells. The idea of using multiple interdependent electromagnetic emitters has led into a novel investigation of complex configurations using specific geometric orientations of multiple electrodes generating electromagnetic fields with precise phase orientation and relationships, which may lead to even more significant coupling with biological systems.

**Figure 2.**
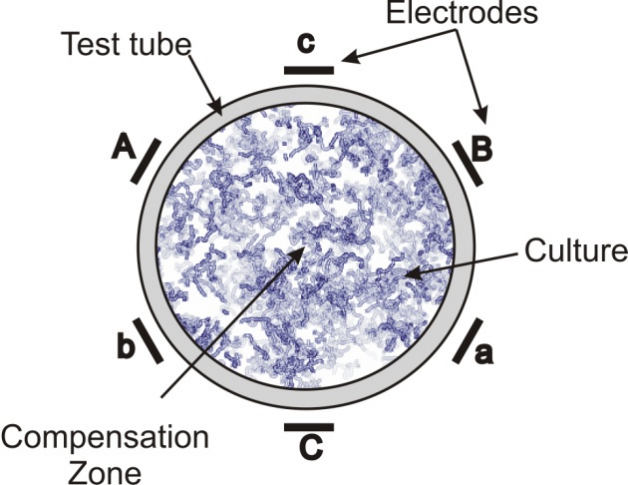
Cross-section of a test tube and a 6-polar electrode configuration for biostimulation of *E.coli.*

**Figure 3.**
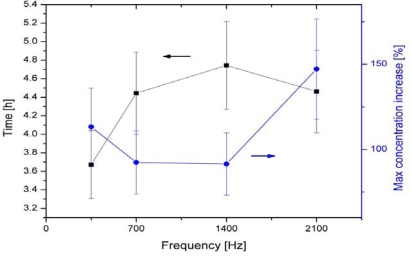
Maximum growth increase, achieved in *E. coli* cultures in test tubes versus frequency of the 6-polar AC EMF treatment (right vertical axis). The left vertical axis shows time to achieve the maximum, while the right axis shows concentration increase with respect to the control.

The interdependent Multipolar (MP) electromagnetic systems were devised and developed by Lensky [63], and Zavalin and his co-workers [13,14]. The MP system may contain a variety of number of poles, *i.e.*, 2, 3, 5, 6, 9, 12, in the symmetrical electrode configuration (C_n_, where n = 2, 3, 5, 6, 9, 12 correspondingly, in notation of the crystallographic groups of symmetry) and complex driving system of interdependent multidimensional transformers that is of most importance. For research with biostimulation of microorganisms, preliminary studies by Zavalin have found that six-pole systems are most effective for microorganisms compared to other configurations. The MP system used in their research consisted of six electrodes in a symmetric hexagonal geometric arrangement (group of symmetry C_6_), driven by a hexapole interdependent transformer system, powered by an amplified function generator. The frequencies of the EMF oscillations are lower than 100 kHz, providing the near-field regime of the MP EMF during the treatment. The MP EMF generated is fine tuned such that the superpositional field, composed of oscillating electric fields from each electrode in the near-field regime undergoes complete destructive interference with a resultant zero-vector electric and magnetic field within a certain area, located near the center of symmetry and called the “compensation zone”. The compensation zone can produce a “breathing” mode where all coils are energized simultaneously to achieve the multipolar compensation zone. A scheme for the 6-polar EMF treatment for the test tube culture studies is shown in Figure 2. The multiple pole EMF configurations have a substantial effect on growth of microorganisms. Maximum achieved growth or gas production increases up to approximately 200% (see Figure 3) were observed in various bacteria, yeast, and protozoa under a 5 or 6-pole configuration at 1 kHz [13], 60 Hz, 0.35–2.1 kHz [14]. The AC voltages at the electrodes were applied 180 degrees out of phase for each opposing set of electrodes, resulting in rather pulsating than a rotating EMF pattern. Figure 3 shows maximum increase in growth of *E. coli* cultures in test tubes under treatment at different frequencies of 6-polar AC EMF. In the plot a maximal achieved ratio of concentration of stimulated *E. coli* culture to concentration of control *E. coli* culture at the same conditions is shown in the right vertical axis. A corresponding time, required to achieve such a maximal relative stimulated increase is shown in the left vertical axis. It should be noted that a depression in growth was observed in 2 and 4-pole system at the similar parameters of the EMF at each electrode. The stimulatory effect was greatest in the lag and log phases of the growth curve. These studies show great promise considering the uniform frequency being emitted was chosen arbitrarily and are open for future research on the optimization of output signal for growth stimulation. The results of studies conducted by Lensky and Zavalin indicate that higher topological EMF, having specific group of rotational symmetry is biologically active. This phenomenon has been previously observed using other types of self-cancelling coil windings [64–66] although the groups of symmetry have not been disclosed. Preliminary evidence indicates that these non-classical designs may be more effective at delivering vibrational information by coupling with interdependent harmonic oscillating cells because these methods produce relatively large biological effects experimentally [13,14,66]. Thus, the multipolar configuration is a strong prospect for exhibiting unique and distinct biological effects.

## 4. Mechanism of Electromagnetic Effects

**Figure 4.**
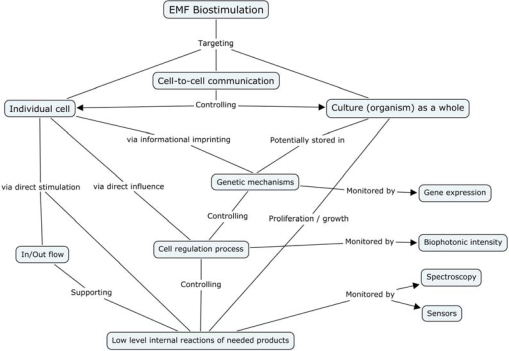
Concept map of an EMF biostimulation at different levels of living systems.

Above observations show growth stimulation by magnetic treatment in a diverse array of organisms (from prokaryotic to eukaryotic) and a variety of stimulative responses by each organism under varied conditions of treatment and growth. While former indicates at some general mode of mechanism(s), the later gives an impression in contrast to it. Lack of adequate information eludes a consensus on the mechanism(s). Several factors appear to be affecting the stimulation process. The flux generating system, intensity of the flux, type of the flux (oscillatory or static), orientation of magnetic poles, duration of exposure, cell density and cell environment (for example type of medium and its ingredients) and other physicochemical conditions affect the process of biostimulation through electromagnetic forces. It has also been marked that the results sometimes do not show repeatability at other locations suggesting that local geomagnetic realities might also affect the process of stimulation. There are physiological effects other than growth that have been observed. These are processes such as carbon uptake, sugar synthesis and oxygen evolution in photosynthesis, synthesis of pigments (chlorophyll, carotenoids and phycocyanins), carbohydrates and proteins, accumulation of micro and trace metals and essential amino acids, fermentative activity and even genetic processes like transposition. They can be stimulated under specific conditions adopted in the experiments. Only one study [29] specifically referred to lipids reported a decline in lipid content under the particular set of treatment. It may be worth noting that an exposure to surprisingly low levels of exogenous electromagnetic fields can have a profound effect on a large variety of biological systems [1]. A number of mechanisms have been proposed for observable magnetobiological and bioelectromagnetic effects at different levels [51]. A concept map, demonstrating different levels of the EMF influence is shown in Figure 4

### 4.1. Ionization and Free Radical Release

Magnetic fields cause oxidative stress in organisms by altering energy levels and spin orientation of electrons and concentration and lifetime of free radicals, which change the relative probabilities of recombination of other interactions with possible biological consequences [67]. Oxidative stress due to the radical pair mechanism becomes applicable around 1 mT which can be common in industrial or laboratory settings, while the geomagnetic field intensity stays below 0.07 mT. Studies with *Chlorella vulgaris* demonstrated that hydroxyl ions increase in magnetically treated medium suggesting alteration of free radical levels in the medium that might hyperactivate antioxidant defense system of the organism. This situation also affects the membrane permeability and ion transport process and might be responsible for the acceleration of chlorophyll excitation by the light [30].

### 4.2. Electrochemical Models

These models explain biological processes considering electromagnetic fields as modulators of molecular information transfer. It is considered that the EMF either itself acts as signal(s) and/or intercepts or modifies the processes of molecular interaction.

#### 4.2.1. Ion Cyclotron Resonance Concept

Many authors have developed the idea of ion cyclotron resonance (ICR) of specific ions like Ca^2+^ and Na^+^ [68] which predicts ELF magnetic effects at the cyclotron frequencies and there harmonics. Later, it was modified to the ion parametric resonance (IPR) model, which includes the cyclotron sub harmonics. The IPR is composed of a number of theoretical models based on classical and quantum electrodynamics where biomagnetic effects are considered as magnetically modulated ion binding in ion-ligand interactions [69]. Free ions move with the cyclotron frequency in a static magnetic field and can be influenced by ELF magnetic fields or appropriate frequencies [70]. The main focus of these studies was the essential role of Ca^2+^ ions in magnetobiology experiments. It is proposed that ion behavior in channels like the acetylcholine receptor have constrictions in them, which cause thermal collisions. Under certain magnetic field parameters the wall collisions could be avoided at certain amplitudes and frequencies determined for the Lorentz force equation [71]. Under these conditions, the ions are predicted to “fly” through the channel unimpeded increasing the membrane permeability. ICR allows circulation of ions through selective enhancement, which affects the rate of biochemical reactions [72].

The fact that magnetic fields can modulate enzyme activities *in vitro* is a crucial observation, because it indicates that enzymes may function as magnetoreceptors [69]. EMF modulations could also initiate changes in the distribution of protein and lipid domains in the membrane bilayer, as well as conformational changes in lipid-protein associations [1]. The interface between cell membrane and extra and intercellular fluids can be electrified on the order of 10^6^ to 10^10^ V cm^−1^ [73]. The impact of an electric field on a biological cell membrane and its change with time may constitute a relevant mechanism of information transmission influencing the membrane properties. The electric field, mainly generated by ions flowing to the membrane from the external environment, can change the molecular distribution of electronic charge inside each lipid molecule, producing perturbations of collective excitations in the mechanical and electrical properties of the lipid chain which can be treated as a mechanism for intermembrane communication, analogous to a damped harmonic oscillations [74].

Pilla *et al.* [73] presented a working model of electrochemical information transfer by which the injection of low-level current can provide functional selectivity in the kinetic modulation of cell regulation. His theory was based on ion/ligand binding being a possible transduction mechanism for the detection of exogenous EMF’s at the cell membrane [75]. In order to derive the specifications for electromagnetic field signals having optimal biological effects, it is first necessary to develop a model for the underlying biological processes which are assumed to be complex physical systems that may be modeled mathematically as non-linear, time-varying, finite-dimensional dynamic systems. They developed a method for the systematic analysis of electrical impedance for each relevant electrochemical pathway of a cellular system [62]. The electrochemical transfer hypothesis postulated that the cell membrane would be the site of interaction of low level electromagnetic fields by altering the rate of binding of calcium ions to enzymes or receptor sites [1]. The Ca^2+^ pathway can be influenced by EMFs on the complex chain of transduction, amplification, and expression. Experimental results have shown that specific ion/ligand binding pathways such as Ca^2+^ binding to calmodulin (CaM) and the ensuing steps of calcium-dependent signaling to intracellular enzymes may act as primary transduction mechanisms for EMF detection leading to an increase in the instantaneous reaction velocity and enzyme kinetics [75,76]. Calmodulin also plays a role in many other important biochemical processes such as cell proliferation, Ca^2+^ membrane transport and plant cell function [77].

An alteration of cell signaling events can lead to changes in cell proliferation and differentiation, which can be initiated, promoted or co-promoted [70]. The capability of the weak EMF to have a bioeffect appears to reside in the informational content of the waveform [1]. The waveform duration and the voltage dependence are the most important parameters to increase the activity of the specific adsorption of an enzyme [62]. The proposed interfacial membrane model reveals that it is entirely reasonable to expect specific electrochemical effects as a result of electrical stimulation with signals of relatively low frequency and amplitude [73].

The incorporation of quantum states into ion interference has also been involved in the explanation of the physical nature of magnetoreception [78]. Variations in magnetic field magnitude affect the phase of ion wave functions and the interference of these phase changes affect the physical observables in quantum mechanics. This theory predicts magnetobiological effects for magnitude/direction modulated magnetic fields, pulsed magnetic fields and weak AC electric fields among others [51]. In these cases, ions of calcium, magnesium, zinc, hydrogen, and potassium appear to be relevant.

However, the most prominent example of a proven bioelectromagnetic mechanism is the radical pair recombination mechanism, which has been demonstrated biochemically *in vitro*. Radical pairs are formed as reaction intermediates in many biochemical reactions within complex reaction chains under the influence of exogenous electromagnetic influence [70]. The recent breakthrough regarding the radical pair mechanism in the blue light receptor protein, cryptochrome, by Schulten and his colleagues, supports the concept that radical pair recombination is involved in magnetoreception in avian navigation. Molecular modeling and calculations showed that the signaling of cryptochrome, which involves a photoreduction process, can be modulated in the presence of a magnetic field on the order of 1 mT inducing an increase in the signaling activity of the protein by ~10% [79,80]. This prediction appears to be consistent with the experimental results on the effect of magnetic fields on cryptochrome-dependent responses in *Arabidopsis thaliana* seedlings attained by Ahmad and coworkers [81]. It is then suggested that the magnetic navigation capability could be mediated by the presence of cryptochrome that is localized in the retinas of migratory birds which could alter how the bird perceives colors enabling something akin to an internal magnetic compass [82]. This radical pair mechanism is probably coupled with the alternative magnetite-based mechanism of magnetoreception and navigation, which poses that the Earth’s magnetic field exerts a minute mechanical force on the magnetite particles found in the upper beaks of migrating birds providing positional information due to fluctuations in the geomagnetic strength in different locations [83].

#### 4.2.2. Stochastic Resonance Amplification

Electromagnetic bioeffects from relatively weak signals are often due to a time-varying electric field, induced by a time-varying magnetic field [1]. However, the ability of weak oscillating EMF fields to interact with living cells has been a source of controversy since thermal and other noise poses restrictions to the detection of weak signals by a cell. Activation of signal pathways by external stimuli connects the physical interactions of the applied EMF to the biological response [70]. In nonlinear systems such as biological sensory apparatus, presence of noise can actually enhance the detection of weak signals, called stochastic resonance [84]. Very small changes in the underlying non-linear kinetics caused by very weak coherent signals and noise can lead to strong, but reversible alterations in the internal nonlinear processes and associated biological function such as ELF influences on G-protein activation dynamic, magnetic field influence on radical pair recombination reactions and weak signal amplification by stochastic resonance incorporated within the Ca^2+^ signal pathway models [70]. The mechanism of stochastic resonance has shown an amplification factor that may exceed a factor of 1,000. This is because in a nonlinear system, the reaction to an external signal may be much greater when acting as a whole than the response of the system’s individual elements. This resonance manifests itself by the appearance of sharp peaks in the power spectrum of the system at the driving frequency and in some of the higher harmonics. Currently, the cell membrane is considered the most likely cellular site for interactions with EMF’s and the possible role of ionic channels of the membrane in the amplification process. The potential well-like structure of an ionic channel makes it the ideal system for stochastic resonance amplification [85].

#### 4.2.3. Long Range Molecular Organization

The application of the nanosized voltmeter, used to measure the electric fields throughout the interior of cellular structures, has indicated that the theoretical calculation of electric field penetration into a cell’s cytosol arising from the membrane and mitochondrial potential do not match the empirically measured values. It is proposed that this may be due to the traditional model using saline solution to simulate the physical properties of the cytoplasm, where alternatively the cytoplasmic structure has been described as having a complex gel-like composition [86,87]. One such possibility for a heterogeneous substance with distinct microdomains is liquid crystal. Liquid crystals are phases of matter that are exhibited by anisotropic organic materials as they undergo cascades of transitions between solid and the liquid states [88]. These mesophases possess symmetry and mechanical properties of long-range orientational order intermediate between those of liquids and of solid crystals. Liquid crystals can undergo rapid changes in orientation of phase transition upon electric or magnetic exposure, or changes in temperature, pH, pressure, hydration, and concentrations of inorganic ions. These properties are ideal for organisms, and it has been found that lipids of membranes, DNA in chromosomes, all proteins, especially cytoskeletal proteins are liquid crystalline in nature [89]. Ho’s group observed that electrodynamic activities might be acting on endogenous non-equilibrium electrodynamic processes involved in phase ordering and patterning domains of liquid crystals [65]. Their findings support that organisms are polyphasic liquid crystals where different mesophases may have important implications for biological organization and function [90].

#### 4.2.4. Josephson Semiconductor Model

From a geometric perspective, it is possible to compare two dividing cells in living systems with a Josephson junction of superconductivity [91]. The Josephson junction may represent a gap junction between two nearby cells coupled via electromagnetic interactions, which provides a mechanism for the transfer of correlated charged particles, electrons, and ions. The gap junctions serve to transmit electrical signals between adjacent cells without the need for mediation by a neurotransmitter or messenger substance [92]. Positive experimental results were attained in yeast cells by examining their current-voltage characteristics and radiofrequency oscillation spectra during cell division [91].

#### 4.2.5. Protein Symmetry

The macroscopic ordering displayed in living systems is an “emergent” property arising from a collective behavior of the elementary microscopic components [93]. The low-frequency internal motions in protein molecules play a key role in biological functions where it is suggested that there is a direct relationship between low-frequency motions and enzymatic activity [94]. The symmetry of protein molecules is also a very important factor in understanding its structure and function, which depends on stability, number of subunits, and folding efficiencies that limits the functionality of the protein. The functionality requirements of symmetry and asymmetry can drive the evolution of proteins to have any of the crystallographic point groups [95]. The breathing motions demonstrated by protein molecules are oscillations of the protein’s symmetry emanating from the center of symmetry of the molecule. These vibrations could potentially be a source and receiver of multipole EMF. Symmetrical and oscillatory nature of proteins, which constitute enzymes, exhibits unique features that have the potential for interaction via external multiple EMF coupling.

#### 4.2.6. Physical Signals in Intermolecular Communication

Progress to understand the intercellular interactions of microorganisms has been linked to the investigation of prokaryotic signaling molecules; however, there is increasing evidence of physically mediated communication for some events, including cell division, adaptation and stress conditions [96]. The hypothesis that electromagnetic forces have a fundamental role in organization and transport of entities is supported by indirect and direct measurements of the electromagnetic fields around living cells.

#### 4.2.7. Electromagnetic Cell Functions

The electromagnetic fields serve as mediators for the interconnection of the organism with the environment as well as between organisms. Electric dipole and multipole moments are common to every biological structure and macromolecule. Oscillating multipole EMF may be generated as a result of interaction of these dipoles and multipoles with electromagnetic emitters and transceivers [97]. Thus the fields produced by the organisms play an important role in the coordination and communication of physiological systems and informational interactions in addition to energetic interactions which play a significant role [98]. The endogenous physiological EM rhythms control and determine the growth and differentiation of cells and are essential for spatiotemporal organization at the subcellular, cellular and organism level [70]. With the recent development of the “nanosized voltmeter” using a voltage-dependent fluorescent nanosensor (E-PEBBLE), the first complete three-dimensional profiling throughout the entire volume of living cells was accomplished. The results indicated that the endogenous electric fields generated penetrate much deeper into the cytosol and non-membrane regions than previously estimated. These measurements support the picture of an electrically complex environment inside the cell [87].

Ions are the transducers of information in the regulation of cell structure. Modification in the interfacial structure of cell membrane alters its ionic composition and constitutes electrochemical information transfer. This alters biochemical and mechanical transport properties of the membrane that is interpreted by the cell as requiring a change in its function which could trigger specific enzyme activity [62,73]. Thousands of chemical reactions are carried out simultaneously and successively in different cellular compartments and are closely coordinated and linked together. The importance of vibrational coherence in the form of electrical and mechanical oscillations has been proven through the experiments [99]. It has been shown for instance that endogenous electric fields exhibiting coherent behavior can have a dominant effect on directed transport of molecules and electrons such that the probability to reach the target is enhanced in comparison with random thermal motion alone [97].

#### 4.2.8. Quantum Physics and Coherence in Biology

Coherence is a fundamental property of a quantum field in which coherent quanta give rise to an order extending over a long distance within which there is a finite probability of finding the system in this order-related state [100]. It is demonstrated in an organism by the movements that are fully coordinated at macroscopic to the molecular levels [90]. The metabolic functioning of living systems has revealed nanomechanical and electrical oscillations in the frequency range of 0.4 to 1.6 kHz, that were found in the yeast, *S. cerevisiae* using atomic force microscopy. If metabolic function was chemically inhibited, the oscillations ceased. It was concluded that the oscillations were consistent with cellular metabolism of molecular motors and may be part of a communication pathway or pumping mechanism by which the yeast cell supplements the passive diffusion of nutrients and/or drives transport of chemicals across the cell wall [101–103]. Physical signal transmission were also found in bacterial cells, where growth-promoting/regulating phonons or sonic vibrations, were effectively transmitted over a distance of at least 30 cm in air, through 2.5 mm plastic barrier, as well as a 2 mm iron plate to distant cultures [104]. Further, sound waves generated from a speaker at specific frequencies promoted colony formation under non-permissive stress conditions [105].

Remarkably, it has been found that even biological events traditionally considered chemically based, such as the lock-and-key model for olfaction, may actually rely more fundamentally on quantum scale atomic processes of inelastic electron tunneling from the donor to a receptor for critical discrimination [106,107]. For example in photosynthesis, light energy is ultimately transduced into chemical and electronic energy through the apparatus of the photosynthetic reaction center. Here the excitation of a chlorophyll molecule by the photon’s energy initiates a series of charge-transfer processes from the antenna pigments to the reaction center via quantum coherence energy transfer [108]. The first steps are so fast that quantum dynamics of the nuclear motion needs to be accounted for as well as electron tunneling [109]. The wave-like characteristics of this energy transfer can explain the extreme efficiency that allows the light harvesting complex to sample vast areas of phase space to find the most efficient path [110].

Most notably, it was discovered that all living biological systems emit ultra-weak photons, or biophotons, which exhibit very unique physical characteristics during spontaneous emission and delayed luminescence. The hyperbolic decay and oscillations of these electromagnetic emissions or biophotons, in the optical regime have been observed experimentally and are indicative of coherent emission in accordance with multimodal laser theory. Coherent electromagnetic radiation strongly suggests the capacity for electromagnetic pathways in intercellular communication [111]. Groups of molecules cannot emit independently from each other because the distance between cells is smaller than the wavelength of the radiation they emit. Since they are coupled by a common radiation field, they will always be coherent [112]. Inside a coherent region or domain, energy travels in a wave-like fashion, whereas in non-coherent domains the energy propagates in a diffusive manner [72]. This coupling field consists of interference patterns reflecting the structure of the antenna system, *i.e.*, groups of molecules, to which it is feedback coupled. Any field has a coherence space-time in which coherent states may exist by having a region where the phase is defined. Outside this region, the phase information is lost, but within it, the interference patterns are formed and a particle loses its classical pictures. Thus the particles and fields within the coherence region must be considered as an indivisible whole [112]. Gurwitsch first discovered coherent emission of ultraweak luminescence on the tips of onions roots in the 1920’s. Modern interpretations of biophotonics conceptualize organisms as biological lasers of optically coupled emitters and absorbers operating at the laser threshold. A technical systems such as a laser, has a fixed coherence region or volume, while organisms may have a multitude of different coherence volumes, which can exist simultaneously and can overlap and demonstrate dynamic properties. The physical components of an organism is coupled with what can be described as a highly coherent, holographic, biophoton field, which has been proposed to be the basis of biological communication at all levels of organization. The components of the organism are seen to be connected in such a way by phase relations of the field that they are instantly informed about each in real-time. The coherent states appear to be fundamental for biological systems since they enable optimization of organization, information quality, pattern recognition and regulation of biochemical and morphogenetic processes [112]. It has been proposed that enzyme dynamics are an outcome of the coherent electromagnetic structure of living systems. Enzymes exhibit selective interactions with specific molecules which strongly suggest the existence of a coherent medium since the molecules no longer interact through random collisions. Classically enzymes are depicted as chemical polymers, however upon applying quantum electrodynamics (QED) principles an enzyme is projected as a coherent domain of its component monomers bound by electrodynamic as opposed to chemical attraction [72].

In various biophotonic experiments with cultures of the unicellular alga *Acetabularia acetabulum* exposed to variety of influences such as varying salt concentrations, chloroform, and temperature modulation, it was concluded that the delayed luminescence was not solely a function of the primary delayed photochemical fluorescence events of the photosynthetic apparatus. However, it demonstrated global correlations and information about the organization of streaming motility of the chloroplast and the cytoplasmic structure of the cell [113]. The cytoskeleton is an important milieu for providing coherent events being the basis for acoustic/photonic transmission. In established *A. acetabulum* cultures the individual cells form extensive electromechanical interactions where phase boundaries and mechanical tensions play an important role, which may be closely connected with biochemical changes and ultimately in a collective biophoton emission pattern [114].

#### 4.2.9. Bioelectromagnetics for Non-Chemical Communication and Signaling

A long history of extensive research on intercellular communication is found in the literature, which has primarily focused on receptor-based chemical signaling, molecular mechanisms, cell recognition, and cell surface receptors; however very few studies have focused on light-mediated interactions of cells, tissues and whole organisms [115]. Kaznacheyev and colleagues in Russia performed over 12,000 experiments in studying distant intercellular communication from two physically separated living tissues or cultures. They used two hermetically sealed vessels attached to each other via an interchangeable window composed of glass or quartz, where each vessel contained an identical culture. One of the vessel’s cells was treated with a specific toxin, *i.e.*, virus, chemical or radiation, while keeping the neighboring culture physically isolated from it. If a quartz window was used, so as to allow UV in addition to the visible and IR range of photons, approximately 75% of the physically isolated cultures began exhibiting toxin specific morphological stress and cell death 12 h after the directly exposed neighbor. However no effect was found if glass was used in the window to block the UV radiations indicating that biophoton signals passing through the quartz window were responsible for the specific morphological response [116–121]. By implementing a photomultiplier tube (PMT), they observed that normal functioning cells emit a uniform photon flux, while with the introduction of a toxin the radiation flux which intensifies at periodic intervals which depend on the different exposed toxin [120]. The harmonic relationship between the UV, visible and IR bands and their phase orientation has been suggested as a potential mechanism of intercellular communication [122] since the existence of coherent fields gives rise to destructive and constructive interference patterns in the space between living cells [123]. The biocommunication in these mutual interference regions leads to an optimized signal/noise ratio as the wave patterns achieve maximum destructive interference or compensation. Once the coherent superposition of modes of biophoton fields breaks down, one expects an increase in biophotonic emission, which was confirmed by Schamhart and Wijk [124], by examining the delayed luminescence of tumor cells as they lose their coherence and capacity for destructive interference by exhibiting exponential as opposed to hyperbolic decay [123]. The importance of biophotons in inter- and intracellular communication has been further confirmed through many other experiments that have been listed in the Table 2.

##### 4.2.10. Endogenous EMF Modeling

Atoms to molecules to macromolecules, the process of modeling these interactions gets increasingly more complex. Biological systems behave like a macroscopic quantum system [112] therefore quantum mechanics is used to describe them. Modern quantum theory in biology has introduced the non-local property of interconnectedness, where the emphasis is no longer on isolated objects, but on relations, exchanges and interdependences on processes, fields and wholes [136].

The ability to detect, interpret and meaningfully interact with the endogenous bioelectromagnetic systems of living organisms could lead to dramatic advancements in modern biological sciences and engineering applications. However, in the case of biophotonic, distant interaction, and multipolar EMF experiments, where there is a destructive interference of EM signals, it becomes exceedingly difficult to directly measure phase conjugated or completely compensated EM fields in superposition. The decomposition of an electromagnetic field into scalar potential functions [137,138] is a traditional mathematical apparatus to describe EMFs at the complete destructive field interference. A conventional wisdom in engineering is that potentials have only mathematical, not physical significance. For instance, classical electrodynamic theory regards the complete cancellation of two fields as an absence of any field or effect. However, besides the case of quantum theory, where it is well known that the potentials are physical constructs, there are a number of physical phenomena - both classical and quantum mechanical, which possess physical significance as global-to-local operators or gauge fields, in precisely constrained topologies, such as the Aharonov-Bohm and Altshuler-Aronov-Spivak effects, the topological phase effects of Berry, Aharonov, Anandan, Pancharatnam, Chiao and Wu, the Josephson effect, the quantum Hall effect, the De Haas - Van Alphen effect, and the Sagnac effect [139]. In particular, the Aharonov-Bohm effect theoretically emphasized the importance of potentials rather than the force fields [140,141]. It was later experimentally demonstrated that interfering electromagnetic potentials could produce real effects on the phase via the magnetic vector potential (A-field) of charged particle systems even though the magnitude of the *force* field was zero around the charged particles [142]. Due to the relative phase factor of two interfering charges, the scalar field can transfer information, even though there is no transport of electromagnetic energy [143]. Furthermore, it appears that information is encoded as frequencies of alternating magnetic vector potential, and should be possible to control chemical reactions *in vitro* and *in vivo* through the interaction of magnetic vector potential with chemical potential [100].

The mathematics to describe the decomposition of an electromagnetic field or wave into two scalar potential functions was advanced by Whittaker at the turn of the century [137,138], which later became the basis for superpotential theory [144,145]. Maxwell’s linear theory is of U(1) symmetry form, with Abelian commutation relations, but it can be extended to include physically meaningful A–field effects by its reformulation in SU(2) and higher symmetry forms. The commutation relations of the conventional classical Maxwell theory are Abelian. When extended to SU(2) or higher symmetry forms, Maxwell’s theory possesses non-Abelian commutation relations, and addresses global, *i.e.*, nonlocal in space, as well as local phenomena with the potentials used as local-to-global operators [139]. Success has been achieved in developing theoretical models for topological criteria for multiple coupled oscillators and higher group symmetry manifolds based on both classical and quantum electromagnetism to explain several phenomena in microbiology, nanoscience and metamaterials [146–150].

The application of these extended, higher topological mathematical models and quantum theories into biophysics and biophotonics may help elucidate the embedded or internal dynamics of the scalar potential functions that comprise the electromagnetic fields that destructively interfere between coupled biological systems or cultures. Despite the overwhelming complexity of modeling interdependent coherent electromagnetic interactions in complex biological systems, there exist both theoretical and empirical evidence that establishes spatial and temporal topology of fundamental geometric superposition, and interdependent relationships, such as multipolar influences, can uniquely affect biological systems.

##### 4.2.11. Role of Water

Water is well known to be an anomalous substance and plays a great role in living organisms. Due to the critical role water plays in biochemical and biological reactions, many studies have focused on the effects of magnetic and electromagnetic fields on water molecules [51]. These experiments have shown that water previously exposed to electrical, magnetic, electromagnetic, acoustic or vibrating fields keeps the acquired biological activity for extended periods of time [151]. Liquid water is clearly a very complex system when considering the complexity of molecular clusters, gas-liquid and solid-liquid surfaces, reactions between the materials and the consequences of physical and electromagnetic processing [152].

The investigation of indirect magnetic field effects have shown that magnetically treated water has changes in light absorption, specific electrical conductivity, magnetic susceptibility, Raman spectrum, index of light refraction, surface tension and viscosity. The exposure of water to a static magnetic field is connected with the energy influence of the field on the water and biostructures. Markov [153] has also shown that static magnetic fields influence the speed of protoplasm movement, the miotic activity, and the quantity of pigments such as chlorophyll *a*, *b* and organic acids in plants. Water stores and transmits information concerning solutes, by means of its hydrogen-bonded network. The conditioning of water via permanent magnetic and electromagnetic oscillating fields has been found to be stimulatory or inhibitory depending on the residence time of the water. *S. cerevisiae* exhibited the strongest influence by measuring a growth rate increase of ~60% after exposing the culture media to 15–30 seconds of a 100 kHz EMF at 2 μT. Longer exposure times that were inhibitory, could become stimulatory after dilution suggesting the existence of active agent(s) generated by the field exposure. Increases in toxicity after applying a biocide compared to a biocide+EMF indicates an enhanced cell wall permeability [154].

Ultra high dilutions are special preparations of a specific compound dissolved in a medium (usually water) that undergo dramatic dilutions (usually thirty 1:100 dilutions) that exceed Avogadro’s number such that the final dilution is void of any original dissolved molecules. Each dilution step is accompanied by some activation force, usually mechanical succussion (shock wave) or vigorous mixing. However, other experiments have used sonication, high-voltage electromagnetic pulses, passive or active resonant circuits. The experimental results indicate that “pure” water samples can retain specific information regarding a “donor” substance which can be quantitatively measured via thermoluminescence, delayed luminescence, excess heat-of-mixing/microcalorimetry, changes in pH and conductivity, alterations to FTIR spectra, enzymatic activity, and modulation of chemical, biochemical, and biological processes usually in accord with the donor substance. These experiments have been carried out with biological bioassays with dinoflagellates comparing succussed media, and modulation to Ca^2+^ channel affinity by non-thermal microwave exposure, as well as investigating physico-chemical effects on purely chemical systems using ultra-high dilution of lithium chloride, sodium chloride, mercuric chloride, and mercuric iodide [155–168].

It has been proposed that the water molecules respond to incident EMF exposure and form metastable water states [164]. The experiments with thermoluminescence, microcalorimetry, and conductivity measurements indicate molecular cluster formation, most likely originating from the hydrogen bond network. The evolution of these physico-chemical parameters with time suggests a trigger effect on the formation of molecular aggregates following the potentization procedure [159]. The various initial perturbations initiate development of a set of chain reactions of active oxygen species in water. Energy in the form of high-grade electronic excitations is released in reactions, which can support non-equilibrium state of an aqueous system [169]. Within these solutions, the molecular aggregates or clusters consisting of water molecules are connected by hydrogen bonds, in far from equilibrium conditions, which can remain in, or move away from their unstable equilibrium state dissipating energy from the external environment in the manner Prigogine has described “dissipative structures” [170]. The lifetime of a particular cluster, containing specific water molecules will be not much longer than the life of individual hydrogen bonds, *i.e.*, nanoseconds, but clusters can continue forever although with constant changing of their constituent water molecules [152]. However, the primacy of hydrogen bonds for the molecular aggregate structures is not essential, as the formation of H-bonded molecules are considered coherence domains in water by Coherent Quantum Electrodynamic Theory, where the H-bond dynamics are transferred to the origin of their pair potentials interacting with zero-point fluctuations of the A-field [171]. The existence of these physicochemical and biological effects from water should elevate water from its traditional role as a passive space-filling solvent in organisms, to a position of singular importance, the full significance of which is yet to be fully elucidated [143].

## 5. Electromagnetic Applications for Production of Algae Biofuels

The application of exogenous electromagnetic influences has been used for various commercial applications and an overview is given in Table 3.

The electric field pulses, or electroporation, have been traditionally implemented in metabolic engineering for gene transformation. Direct electroporation of a cyanobacterium *Synechococcus elongates*, introduced the enzyme Clostridial hydrogenase, which may lead to the development of a variety of hydrogenases for hydrogen production, coupled to photosynthesis in cyanobacteria for bioenergy production [186]. In addition to membrane-permeabilizing effects, it can also induce biochemical and physiological changes in plant protoplasts, such as stimulating protein and DNA synthesis, and cell division and differentiation [187]. Alternatively, electroporation can also be used as a process for cell membrane modification for enhanced oil/lipid extraction from microalgae for biodiesel. A preliminary study found 20% increase in oil yield, shorter extraction time, and 2/3 less solvent used without affecting the composition of extracted fatty acids compared to chemical solvent alone [33].

Although most electrochemical and electromagnetic effects mentioned thus far have been focused on biological responses, an integrated biorefining system also requires process engineering technologies for harvesting algae for instance. An electrochemical process using direct electric current, called electroflocculation, has a long history as a wastewater treatment technology for solid/liquid, and liquid/liquid separation [188]. This technology combines the use of a sacrificial electrode that dissolves to coagulate suspended particles (electrocoagulation) along with the use of electrolysis, which produces H_2_ microbubbles that float the aggregates or flocs to the surface (electroflotation) for easy removal from the water. Electroflocculation is a promising technology for harvesting microalgae biomass since it has several advantages over other conventional processes. The efficiency of particle/biomass separation in electroflocculation is over 90% and this technology does not require moving parts, and consumes relatively little energy (0.3 kWh m^−3^) with substantially lower capital costs [189]. In fact, a more recent study shows a 99.5% removal of total suspended solids (TSS) and chlorophyll *a* (algae) by applying 0.55 kWh m^−3^ for 15 minutes [190].

Bioelectrochemical denitrification is a novel technology being used for the treatment of ammonium and nitrate-containing wastewater by means of denitrifying bacteria and hydrogen gas produced on the cathode by the electrolysis of water. The denitrifying microorganisms are usually immobilized as a biofilm on graphite or a stainless steel cathode. A nitrate removal efficiency of 98% was observed at 20 mA when phosphate was used as a buffer. The studies suggested that the application of bioelectro-reactors could be used for reduction and oxidation treatments of ammonium and nitrate-containing wastewaters [191–193].

In many cases, real-time monitoring of cultures is critical for productive and efficient cultivation/fermentation in which the optical density, pH, and dissolved gas levels may not elucidate the underlying bioprocesses occurring, especially when evaluating electrochemical or electromagnetic interactions. Pulsed Amplitude Modulation Fluorometry or PAM fluorometry is a special method for measuring fluorescence from photosynthetic organisms for real-time culture monitoring of the photosynthetic apparatus. It uses the characteristics of the fluorescence emitted by chlorophyll *a* as a probe for the biophysics and biochemical events occurring in the electron transport chain of Photosystem I & II. These measurements are a unique indicator of photosynthesis and provide information about the maximum photosynthetic efficiency (by a dark-adapted sample), the effective photosynthetic efficiency (under constant illumination), and the non-photochemical quenching (heat dissipation). These parameters indicate what fraction of the photon energy absorbed by the organism is used for photochemistry, dissipated as heat, and re-emitted as fluorescence [194]. Papazi and his colleagues found that PAM Fluorometry in conjunction with traditional biomass analysis was able to show how extremely high CO_2_ concentrations impacted the photosynthetic apparatus, which stimulated intense biomass production in the microalgae, *Chlorella minutissima* [195].

The use of the biophotonic method of delayed luminescence (DL) has been used for quality control applications with fruits and vegetables. A study with tomato fruits revealed marked changes due to different harvesting maturities. It was found that tomatoes exhibited DL measurements related to color and respiration as well as significant differences in soluble solids content and dry matter percentage. Therefore, DL values are directly related to tomato harvest maturity. Qualitative traits can depend on harvest maturity, thus suggesting that delayed luminescence could be used as a nondestructive indicator of fruit quality [196].

In addition to fluorescent measurements, the fast, non-invasive measurement of biological cells by dielectric spectroscopy, or impedance spectroscopy, is currently being utilized to determine cellular parameters, such as living cell volume, cell number distribution over cell cycle phase, cell length, internal structure, complex permittivity, and intracellular and extracellular media and morphological factors. The electrical and morphological properties of the cell membrane are assumed to represent sensitive parameters of the cellular state [197]. It has been demonstrated to be a powerful method for dielectric monitoring of biomass and cell growth in ethanol fermentation and the extension of the scanning dielectric microscope is a promising tool for dielectric imaging of biological cells [198]. The real-time monitoring of yeast cell division by measuring the dielectric dispersion can enable to tracking of cell cycle progression using an electromagnetic induction method [199]. Recently, online monitoring of lipid storage in microorganisms (yeasts) was conducted which found that using dielectric spectroscopy data, the change in capacitance divided by the characteristic frequency being used showed a clear shift from the growth phase to the lipid accumulation phase, which could be of use for technical control of intracellular biopolymer or oil accumulation, as well as enzyme overproduction [200]. Moreover, it has been established that there exists a connection between D.L. and impedance spectroscopic parameters, which explore related structures and mechanisms in living samples [201]. By applying the knowledge, gained from biophotonic and bioelectromagnetic experiments, it may be possible to detect, interpret and interact with the endogenous coherent electromagnetic signals that are correlated with regulation, communication, and organization of biological systems since oscillation dynamics are of essential importance in intercellular and intracellular signal transmission and cellular differentiation [70]. These signals may initially give us real-time insight into the internal dynamics of an organism or culture, which may precede the physically/chemically observable events.

Induction of specific cellular response to biophotonic signals could perhaps be achieved to stimulate a desired biological effect such as enhancement of lipid or enzyme synthesis or metabolite modulation using electromagnetic fields instead of an external stress or a biochemical initiator.

Electromagnetic bioprocesses such as electroflocculation and electroporation can be used for algal harvesting and biomass processing. The use of static and oscillating electromagnetic fields has a potential for the enhancement of cell proliferation, metabolite production and cell cultivation for biomass production. After extraction, fermentation of the algae feedstock, using applied electric field parameters, can be designed for enhanced substrate utilization and higher ethanol/butanol yields. Any residual biomass may then be used for enhanced production of methane from anaerobic digestion using specific frequencies of microwaves reported by Banik *et al.* [5], who showed how the EM exposure parameters could be used for potential bioenergy/biofuel applications.

The application of electromagnetic coupling to electrochemical biological pathways, which have been studied and commercialized for biomedical applications can be introduced into bioengineering. Here investigations into the electrochemical impedance properties for triggering biochemical cascades of desired signaling pathways in microorganisms for bioenergy applications deserve significant attention. The application of exogenous EMF influences may synergistically couple with endogenous electric fields for enhancing directed mass transport in cells. It is conceivable that any cell could be stimulated, inhibited, or made to exhibit passive response, depending upon the appropriate choice of frequencies and amplitudes of the excitation signals employed [62]. The induction of mitosis for cell proliferation, as well as the stimulation of enzymatic pathways associated with energy metabolism and storage such as lipid accumulation, needs modeling and more experimentation. Such electrochemical processes may also be relevant for accelerating enzymes, such as Rubisco, in the carboxylation pathway of photosynthesis to enhance specific binding of CO_2_ and limiting photorespiration to enhance overall system efficiency in microalgae or plants. The greatest challenge may be the evaluation of the proper dosimetry for modulation of the desired biochemical cascade [1].

The introduction of the complex topology of multipolar electromagnetic fields may provide an enhanced coupling effect to complex, interdependent biological systems. Such systems may be tailored to uniquely control endogenous electromagnetic processes and communication for cellular functioning and organization. Furthermore, the bioproducts, generated by engineered multipolar hybrid biosystems have additional properties. For example biofuel/bioenergy production processes potentially can have higher productivities through better substrate utilization and conversion and shorter processing times.

The use of electrochemical/electromagnetic triggering of specific metabolic pathways could be coupled with biophotonic analysis, where rapid screening and fine tuning of a desired effect could be devised. Such bioelectromagnetic and biophotonic monitoring could also be of significant interest to metabolic and genetic engineering, by incorporating and correlating electrochemical and endogenous electromagnetic signals with gene expression and enzymatic activity.

The pervasive utilization of water in the cultivation of microorganisms particularly with algae, suggests possible application of the principles discovered in ultra-high dilution and activation studies to enhance and modulate biological responses. Water used in the growth medium for cultivation may be imprinted by various methods (electromagnetic information transfer probably being the most convenient) with specific information on relevant organic and inorganic nutrients as well as biochemical growth promoters to enhance growth characteristics, while decreasing demand for the potentially large amounts of the donor substance.

**Figure 6.**
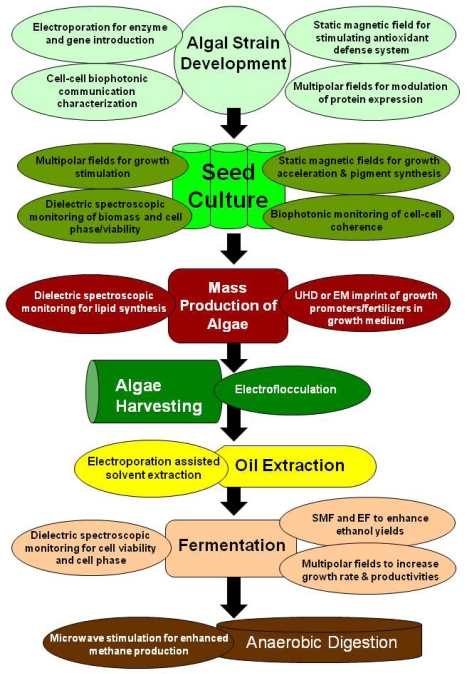
Integrated biostimulation/biofuel production system.

**Figure 7.**
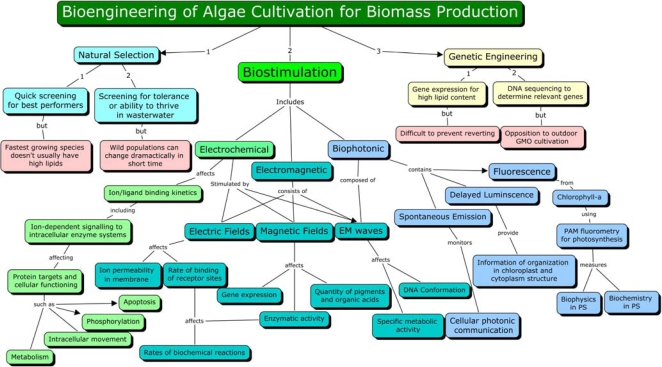
Bioengineering of algae cultivation.

The combination of these separate disciplines, could blossom into a new integrative bioengineering approach that incorporates the diverse specializations of molecular biology, biochemistry, electrochemistry, biophysics, and quantum physics that could open up significant biotechnological progress of engineering of living systems for bioprocessing, bioconversion, biofuel and bioenergy applications (Figures 6 and 7).

## 6. Conclusions

Traditional cultivation and manipulation of biological systems have consisted of natural selection and genetic engineering modalities. Recently metabolic engineering and synthetic biology are gaining wide attention from the scientific community due to their immense potential in altering the metabolism in living systems especially microbes for medical, agricultural, industrial and environmental applications. However, genetic manipulation of microbes and living systems for agricultural and environmental applications may affect the ecosystem adversely as the changes in the species are permanent and inherited. In case of bioelectromagnetic stimulation, system reacts more in a transient fashion. The changes even if inherited are not sustained by the species for long thus they might be safer over genetic manipulation.

This review provided a broad spectrum of potentially useful bioeffects on microorganisms that are currently or potentially valuable in biotechnology and bioenergy. At this point it is difficult to ascertain exactly how economically feasible these emerging methods and potential technologies will be due to a variety of unknown factors from the nature and scalability of the bioeffects to the electronic design and efficiency for large-scale implementation. However, it is the aim to stimulate interest in the field and invite scientists with new ideas into the long standing discipline of bioelectromagnetics that modern biology is only recently starting to understand. In the new horizon of biologically derived fuels and materials, advancements in the area of biostimulation could impact the direction of biotechnology towards an energetic approach that may boost the potential for emerging biotechnologies such as microalgae based biofuel and biomass production.

Biofuels, bioenergy and carbon capture are considered to be the current priorities for the entire global community. The International Energy Agency (IEA) has reported that the world’s primary energy need is projected to grow by 55% between 2005 and 2030, at an average annual rate of 1.8% per year. Fossil fuels are the main source of primary energy and if the governments around the world stick to current policies, the world will need almost 60% more energy in 2030 than today. Transportation is one of the fastest growing sectors using 27% of the primary energy. At the present staggering rates of consumption, the world’s fossil oil reserve will be exhausted in less than 45 years. Considering the negative impacts of utilizing fossil fuel energy sources, many countries have already mandated the use of biofuels and set the targets to replace significant quantities of fossil derived fuels. Second and third generation biofuels such as lignocellulosic ethanol and algae biofuels are considered to be the viable alternatives as they do not compete with food needs. Bioelectromagnetic stimulation of microbes particularly with microalgae provides a new extended domain of disciplines and methodologies for cultivation, harvesting and processing of biomass for production of biofuels, bioenergy and added value bioproducts. Though this technology is promising, lots of research efforts are needed in future to exploit its commercial potential for biotechnology and biofuel applications.

## Figures and Tables

**Figure 5.**
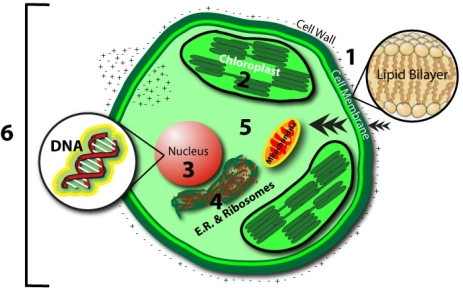
Molecular interaction sites of electromagnetic influences.
**Cell Membrane**
- Magnetic field oscillations may increase membrane permeability under ion cyclotron resonance- Increased circulation and selective enhancement of ion flow may affect the rate of biochemical reactions- Alter the rate of binding of calcium ions to enzymes or receptor sites- Change distribution of protein and lipid domains, and conformational changes in lipid-protein associations- Change internal molecular distribution of electronic charge inside lipid molecule in the membrane bilayer- May play the primary role in the stochastic resonance amplification process**Chloroplast**
- May modulate the quantity of pigments, such as chlorophyll, phycocyanin, and beta-carotene**Nucleus/DNA**
- Magnetic field affects specific gene expression- Individual DNA sequences may function as antennae- Leads to changes in DNA conformation- May activate different DNA sequences depending on field intensity- Can affect enzyme activity**Proteins:**
- Breathing motions are the source and receiver of multipole EMF- Potential coupling mechanism for external multipolar influences**Protoplasm**
- Static magnetic fields influence the speed of protoplasm movement, miotic activity, and quantity of organic acids in plants**Whole Cell**
- Biophotonic emission and interaction with nearby cells- Endogenous electric field modulation may alter natural processes

**Table 1.** Summary of electromagnetic treatments of some microorganisms.

**Organism**	Class*	**EM Intensity**	**Biological effect**	**Reference**
**Archaea**				
*Methanosarcina barkeri*	MW	13.5–36.5 GHz	Increase in growth, cell count and size and methane production	[5]
**Eubacteria**				
	PMF	0.05–1 mT	Stimulated transposition activity & reduced cell viability	[6]
	AC MF	16, 60 Hz	Enolase activity stimulation; Suppression of enolase activity	[7]
		0.05–1 mT	Reduced transposition activity & enhanced cell viability	[6]
*E. coli*	OMF	100 mT	Exposure time dependent stimulation or inhibition of cell viability	[8]
		30 μT	Cell density dependent changes in AVTD	[9]
	DC EF	NA	Increase in growth, removal of inhibitory compounds in medium	[10]
	AC MF	0.1–1 mT @ 50 Hz	Significant morphotype changes & alteration during cell division	[11]
	ACEF	2.5–50 V/cm @ 0.05–100 kHz	Stimulation of membrane bound ATP synthesis, optimum at 100 Hz	[12]
	6-polar ACEF	0.35–2.1 kHz for test tubes 60 Hz for Petri dishes	Increase in growth in test tubes (147 ± 24%) and colonies (42–179%)	[13,14]
*Bacillus cereus*	6-polar ACEF	1 kHz	Increase in growth in tubes (196 ± 29%) and colonies	[13,14]
*B. mucilaginosus*	SMF	~0.39 T	Increase in growth	[15]
*B. subtilis*	AC MF	0.8, 2.5 mT, 0.8 and 1 kHz	Growth increase and interestingly a loss of intercellular cohesion	[16]
*Paper to be seen*	AC MF	0–0.3 Hz @ 5−90 mT	Elevated or even diminished growth rates for *Bacillus subtilis, Candida albicans, Halobacterium, Salmonella typhimurium,* and *Staphylococci*	[17]
*Pseudomonas stutzeri*	PMF	0.6–1.3 mT	Increase in growth	[18]
*Trichoderma reesei*	PMF	1.5 mV cm^−1^	Increase in growth, cellulase activity and secretion	[19]
*Streptomyces noursei*	PMF	1.5 mV cm^−1^	Increased antibiotic production, O_2_ evolution, glucose uptake	[20]
*Salmonella typhimurium*	OMF	15 mT@ 0.3Hz	Growth stimulation, Mutation reversion rate unaffected	[17]
*Micrococcus denitrificans*	SMF	500–800 mT	Growth inhibition followed by stimulation after 6 h	[21]
*Corynebacterium glutamicum*	AC MF	4.9 mT, 50 Hz	Increase in ATP levels by about 30%	[22]
Natural Flora	SMF	22 mT	Enhanced degradation of phenolic waste liquors	[23]
Natural Flora	PEF	1.25 – 3.25 kVcm^−1^	Enhanced biosorption of uranium	[24]
Bacteria & yeast	OMF	15 mT@ 0.3 Hz	Larger increase (30%) in growth in gram –ve (*Psuedomonas aeruginosa, Halobacterium halobium*) *than gram +ve (Bacillus subtilis, Staphylococcus epidermidis*) and yeast (*Candida albicans*)	[17]
*Rhodobacter sphaeroides*	AC/DC MF	0.13–0.3 T	Increase in porphyrin synthesis, Enhanced expression of 5-aminolevulinic acid dehydratase	[25]
**Cyanobacteria**				
*Spirulina platensis*	SMF	10 mT	Increase in growth (50%), O_2_, sugar, phycocyanin	[26]
		250 mT	Increase in growth (22%), CNP-Uptake, Chl, minerals	[27]
	MW	7.1 mm @ 2.2mWcm^−2^	Increased growth (50%)	[28]
*Anabaena doliolum*	SMF	300 mT	Increase in growth, pigments, carbohydrate and protein	[29]
**Algae**				
*Chlorella vulgaris*	SMF	10–35 mT	Increase in growth (100%); Stimulated antioxidant defense	[30]
*Chlorella* sp.	SMF	6–58 mT	Increase in growth (NA)	[31]
*Dunaliella salina*	SMF	10–23 mT	Increase in growth (90%), and β-carotene	[32]
*Scenedesmus* sp.	PEF	NA	Enhanced oil extraction- Solvent+Electroporation	[33]
**Yeast**				
*Saccharomyces cerevisiae*	PMF	~ 4.7 μT	Increased activity of alcohol dehydrogenase	[34]
	OMF+SMF	20 mT + 8 mT	Increase in ethanol, sugar utilization	[35]
*S. cerevisiae*	OMF	0.28–12 mT	Increase in growth	[36]
	OMF	0.2–12 mT @ 50 Hz	Increase in growth (25 +/− 5%)	[37]
	AC/DC EF	100/10 mA	Increase in growth, organic acid production, cell budding	[38]
	MW	42GHz@ < 3 mWcm^−2^	Frequency dependent increase or decrease in growth	[39]
	6-polar ACEF	1 kHz	Increase in gas production (195 ± 20%)	[13,14]
	AC MF	0.5 μT, 100 200 Hz	30% reduction in respiration	[40]
*Saccharomyces* sp.			Better UV survival in those given magnetic pretreatment	[41]
				[42]
			Respiration stimulation	
*S. fragilis*	SMF	~0.26 T	Increase in growth (27–36%)	[15]
*Kluyveromyces marxianus*	PEF	0.25 kV	Increased ethanol production and cellobiose utilization	[43]
*Physarum polycephalum*	ELF EMF	45,60,75 Hz	Delayed mitosis by 0.5 to 2 h	[44]
	AC MF	0.1 mT, 60 Hz	Lower ATP levels but no decreased respiration	[45,46]
		0.2 mT and 60 and 75 Hz	Reduced respiration	
**Protozoa**				
*Trichomonas vaginalis*	SMF		Field strength dependent growth stimulation/inhibition	[47]
**Ciliophora**				
*Paramecium tetraurelia*	AC MF	1.8 mT, 72 Hz	Ca^2+^ specific increase in cell division rates, absent in the presence of a Ca^2+^ blocker, Alterations in membrane fluidity	[48]
*Tetrahymena pyriformis*	AC MF	10 mT, 60 Hz	Delayed cell division and increased oxygen uptake	[49]

* AC-EF: alternating current electric field; DC-EF: direct current electric field; MW: microwave; OMF: oscillating magnetic field; SMF: static magnetic field; PEF: pulsed electric field; PMF: pulsed magnetic field.

**Table 2.** Overview of biophotonic and distant intercellular interactions (D.I.) experiments, delayed luminescence (D.L.), and spontaneous emission (S.E.).

**Culture**	**Experiment**	**Effect**	**Reference**
*Daphnia*	D.I. & S.E.	Established destructive interference found at natural population density	[125]
*D. tertiolecta*	D.I. & D.L.	Changes in external environment demonstrated dose/intensity dependent decay curves	[126]
*P. elegans*	D.I. w/E-Field	E-field stimulated distant culture’s photonic activity and synchronization	[127,128]
*Gonyaulax sp.*	D.I.	Established destructive interference and synchronization of photon pulses	[129]
XC tumor cells	D.I.	Dense cell culture stimulated growth rate of isolated culture via optical contact	[116]
Epithelial cells	D.I. w/H_2_O_2_	Reduction in protein, increased nuclear activation, and structural damage	[130]
*E. coli*	D.I.	Synchronized growth parameters when in optical contact of Vis-IR.	[96]
*S. cerevisiae*	D.I.	Stimulation of cellular subdivision via optical coupling with culture of same type	[131]
*P. fluorescens*	D.I.	Long range interactions of an isolated culture diminished adhesion between cells of another culture	[132]
*V. costicola*	D.I.	Isolated treated culture stimulated growth of second culture of same species	[133]
Fibroblasts	D.I. w/Viruses	Three viral effects transferred to 72–78% of distant isolated cells	[134]
	D.I. w/HgCl_2_	Effects transferred to 78% of distant isolated cells	
	D.I. w/Rad	UV radiation effects transferred to 82% of distant isolated cells	
*L. pekennisis*	S.E.	Measured coherent emission from 200–800 nm which differed between male and female specimens	[135]

**Table 3.** Overview of existing application of bioelectromagnetic fields.

**Biomedical**	**Influence**	**Application**	**Reference**
	PEMF	Chronic wound healing, and non-union fracture healing	[172]
		Chronic wound healing	[173]
		Treatment of osteonecrosis	[174]
		Treatment of pressure ulcers in spinal-cord injuries	[175]
		Treatment of osteoarthritis of the knee	[176]
		Treatment of grade I & II ankle sprains	[177]
		Treatment of venous leg ulceration	[178]

**Agricultural**	**Influence**	**Application**	**Reference**

	SMF	Treated water to stimulate germination in *Pinus tropicalis* seeds	[179]
		Treated chickpea seeds increased germination, seedling and root length & size	[180]
		Treated water increased plant height, branch number, and shoot dry weight	[181]
		Treated wheat seeds increased germination, yields, and protein	[182]
		Treated rice seeds and water increased rate and % of germination	[183]
		Treated barley seeds and water increased length and weight	[184]
	OMF	Treated tomato seeds for increased growth, yields, and disease resistance	[185]
