# Correction: Hunt, R.W. *et al.* Electromagnetic Biostimulation of Living Cultures for Biotechnology, Biofuel and Bioenergy Applications. *Int. J. Mol. Sci.* 2009, *10*, 4515–4558

**DOI:** 10.3390/ijms10114719

**Published:** 2009-10-30

**Authors:** Ryan W. Hunt, Andrey Zavalin, Ashish Bhatnagar, Senthil Chinnasamy, Keshav C. Das

**Affiliations:** 1 Department of Biological and Agricultural Engineering, The University of Georgia, Athens, GA 30602, USA; E-Mails: bhatnagarashis@gmail.com (A.B.); csenthil@engr.uga.edu (S.C.); kdas@engr.uga.edu (K.C.D.); 2 Mass Spectrometry Research Center, Vanderbilt University Medical Center, Nashville, TN 37232, USA; E-Mail: andrey.zavalin@vanderbilt.edu (A.Z.)

We found some errors in the paper published in International Journal of Molecular Sciences [1]. The table 1 had some typographical errors, information regarding field intensity missing, and the nomenclature did not match the legend. The corrected version is given below.

**Table 1.** Summary of electromagnetic treatments of some microorganisms.

**Organism Class**	*EM	**Intensity**	**Biological effect**	**Reference**
**Archaea**				
*Methanosarcina barkeri*	MW	13.5–36.5 GHz	Increase in growth, cell count, size and methane production	[5]
**Eubacteria**				
*E. coli*	SMF	0.05–1 mT	Stimulated transposition activity and reduced cell viability	[6]
	OMF	16, 60 Hz	Stimulation and suppression of enolase activity	[7]

	OMF	0.05–1 mT	Reduced transposition activity and enhanced cell viability	[6]
	OMF	100 mT	Exposure time dependent stimulation or inhibition of cell viability	[8]
	OMF	30 μT	Cell density dependent changes in AVTD	[9]
	DC EF	NA	Increase in growth and removal of inhibitory compounds in medium	[10]
	OMF	0.1–1 mT @ 50 Hz	Significant morphotype changes and alteration during cell division	[11]
	ACEF	2.5–50 V/cm @ 0.05–100 kHz	Stimulation of membrane bound ATP synthesis, optimum at 100 Hz	[12]
	6-polar ACEF	0.35–2.1 kHz for test tubes 60 Hz for Petri dishes	Increase in growth in test tubes (147 ± 24%) and colonies (42–179%)	[13,14]
*Bacillus cereus*	6-polar ACEF	1 kHz	Increase in growth (196 ± 29%)	[13,14]
*B. mucilaginosus*	SMF	~0.39 T	Increase in growth	[15]
*B. subtilis*	OMF	0.8, 2.5 mT, 0.8 and 1 kHz	Increase in growth and a loss of intercellular cohesion	[16]
*Bacteria & Yeast*	OMF	0–0.3 Hz @ 5–90 mT	Elevated or even diminished growth rates for *Bacillus subtilis, Candida albicans, Halobacterium, Salmonella typhimurium,* and *Staphylococci*	[17]
*Pseudomonas stutzeri*	SMF	0.6–1.3 mT	Increase in growth	[18]
*Trichoderma reesei*	SMF	1.5 mV cm^–1^	Increase in growth and cellulase activity and secretion	[19]
*Streptomyces noursei*	SMF	1.5 mV cm^–1^	Increased antibiotic production, O_2_ evolution and glucose uptake	[20]
*Salmonella typhimurium*	OMF	15 mT@0.3Hz	Growth stimulation; Mutation reversion rate unaffected	[17]
*Micrococcus denitrificans*	SMF	500–800 mT	Growth inhibition followed by stimulation after 6 h	[21]
*Corynebacterium glutamicum*	OMF	4.9 mT, 50 Hz	Increase in ATP levels by about 30%	[22]
Natural Flora	SMF	22 mT	Enhanced degradation of phenolic waste liquors	[23]
Natural Flora	PEF	1.25 – 3.25 kVcm^–1^	Enhanced biosorption of uranium	[24]
Bacteria & yeast	OMF	15 mT@0.3 Hz	30% increase in growth in gram –ve bacteria (*Pseudomonas aeruginosa, Halobacterium halobium*) than *gram +ve (Bacillus subtilis, Staphylococcus epidermidis*) and yeast (*Candida albicans*).	[17]
*Rhodobacter sphaeroides*	OMF/ SMF	0.13–0.3 T	Increase in porphyrin synthesis; Enhanced expression of 5-aminolevulinic acid dehydratase	[25]
**Cyanobacteria**				
*Spirulina platensis*	SMF	10 mT	Increase in growth (50%), O_2_ evolution and sugar and phycocyanin production	[26]
		250 mT	Increase in growth (22%), CNP and minerals uptake and chlorophyll content	[27]
	MW	7.1 mm @ 2.2mWcm^–2^	Increased growth (50%)	[28]
*Anabaena doliolum*	SMF	300 mT	Increase in growth, pigments, carbohydrate and protein	[29]
**Algae**				
*Chlorella vulgaris*	SMF	10–35 mT	Increase in growth (100%); Stimulated antioxidant defense	[30]
*Chlorella* sp.	SMF	6–58 mT	Increase in growth (NA)	[31]
*Dunaliella salina*	SMF	10–23 mT	Increase in growth (90%), and β-carotene content	[32]
*Scenedesmus* sp.	PEF	NA	Enhanced oil extraction	[33]
**Yeast**				
*Saccharomyces cerevisiae*	PMF	~ 4.7 μT	Increased activity of alcohol dehydrogenase	[34]
	OMF+SMF	20 mT + 8 mT	Increase in ethanol and sugar utilization	[35]
*S. cerevisiae*	OMF	0.28–12 mT	Increase in growth	[36]
	OMF	0.2–12 mT @ 50 Hz	Increase in growth (25 +/− 5%)	[37]
	AC/DC EF	100/10 mA	Increase in growth, organic acid production and cell budding	[38]
	MW	42GHz@ < 3 mWcm^–2^	Frequency dependent increase or decrease in growth	[39]
	6-polar ACEF	1 kHz	Increase in gas production (195 ± 20%)	[13,14]
	OMF	0.5 μT, 100*–*200 Hz	30% reduction in respiration	[40]
	SMF	7.28 T	Better UV survival; Stimulation of respiration	[41] [42]
*S. fragilis*	SMF	~0.26 T	Increase in growth (27–36%)	[15]
*Kluyveromyces marxianus*	PEF	0.25 kV	Increased ethanol production and cellobiose utilization	[43]
*Physarum polycephalum*	OMF	45,60,75 Hz	Delayed mitosis by 0.5 to 2 h	[44]
	OMF	0.1 mT, 60 Hz	Lower ATP levels but no decreased respiration	[45,46]
		0.2 mT and 60 and 75 Hz	Reduced respiration	
**Protozoa**				
*Trichomonas vaginalis*	SMF		Field strength dependent growth stimulation/inhibition	[47]
**Ciliophora**				
*Paramecium tetraurelia*	OMF	1.8 mT, 72 Hz	Increase in cell division rates; Alterations in membrane fluidity	[48]
*Tetrahymena pyriformis*	OMF	10 mT, 60 Hz	Delayed cell division and increased oxygen uptake	[49]

* AC-EF: alternating current electric field; DC-EF: direct current electric field; MW: microwave; OMF: oscillating magnetic field; SMF: static magnetic field; PEF: pulsed electric field; PMF: pulsed magnetic field.

We apologize for any inconvenience caused to the readers.
